# Solvent effects on the electronic structure, excited states, and nonlinear optical properties of two anthracene-based chalcone derivatives: a DFT and TD-DFT study

**DOI:** 10.1007/s00894-026-07009-7

**Published:** 2026-10-21

**Authors:** Renato Medeiros

**Affiliations:** Universidade Estadual de Goiás (UEG), Anápolis, Goiás Brazil

**Keywords:** **C**halcone derivatives, Nonlinear optics, TD-DFT, Solvation, First hyperpolarizability, Charge transfer

## Abstract

**Context:**

Anthracene-based chalcones are π-conjugated donor–acceptor chromophores with potential second-order nonlinear optical applications, but solvent-field polarization and solvent-induced geometry relaxation can affect their response in different ways. This work compares ANT–IND and ANT–DMNAF, which differ in donor environment, to identify how the medium controls absorption and static first hyperpolarizability. Solvation red-shifts the principal UV band by about 14 nm and substantially increases the computed nonlinear optical response. Methanol-optimized ANT–DMNAF reaches ${\beta}_{\parallel }=10269 a.u. (88.7\times {10}^{-30} esu)$, exceeding ANT–IND under the same conditions and indicating a solvent-dependent crossover in the computed NLO ranking.

**Methods:**

Gas-phase and solvated geometries were optimized with density functional theory at the CAM-B3LYP/6–31 + G(d,p) level using the SMD continuum model in Gaussian 16. Harmonic frequency calculations confirmed true minima. The six media considered comprise the gas phase plus five solvents: n-hexane, toluene, dichloromethane, methanol, and DMSO. Time-dependent DFT calculations with 30 excited states provided UV–Vis spectra, and static dipole moments, isotropic polarizabilities, and first hyperpolarizabilities were obtained analytically. NLO properties were evaluated with both fixed gas-phase geometries (GasGeom) and solvent-relaxed geometries (SolvGeom); this comparison is used as a computational sensitivity analysis and not as an experimentally separable partition of the response.

## Introduction

Organic molecules exhibiting large second-order nonlinear optical (NLO) responses have attracted sustained research interest owing to their potential applications in photonic devices, electro-optic modulators, frequency doublers, and optical data storage systems [1–3]. Among these, donor -π- acceptor (D-π-A) chromophores, in which an electron-donating group and an electron-withdrawing group are linked through a conjugated bridge, represent the most extensively explored molecular architecture for NLO activity [4]. The efficiency of the NLO response in such systems is governed by the extent of intramolecular charge transfer (ICT) from the donor to the acceptor moiety, which is modulated by the nature of the donor and acceptor groups, the length and planarity of the conjugated bridge, and the surrounding medium [5].

Chalcone derivatives, characterized by an $\alpha ,\beta -$ unsaturated carbonyl bridge, constitute an attractive class of donor -π- acceptor chromophores. Their modular structure allows systematic variation of donor and acceptor fragments, while the conjugated backbone can facilitate charge delocalization [6]. Chalcones and related derivatives can display substantial first hyperpolarizabilities, thermal stability, and synthetic tunability [7, 8]. Incorporation of a polycyclic aromatic fragment such as anthracene can extend the π framework; however, the effective conjugation also depends on the relative geometry between the anthracenyl and enone fragments, because torsion about the connecting bond can modulate orbital overlap along the charge-transfer pathway [9].

A critical aspect of molecular NLO behavior is the influence of the solvent environment. In solution, the surrounding medium can modify the ground-state charge distribution, molecular geometry, excitation energies, and electric-response properties of push–pull chromophores [10, 11]. Previous theoretical and experimental studies have shown that solvent-induced polarization and structural relaxation can substantially alter static and frequency-dependent nonlinear optical responses, including first hyperpolarizabilities, in donor–acceptor polyenes, conjugated organic chromophores, transition-metal complexes, photochromic systems, azo chromophores, and related NLO molecules [12–19]. Continuum calculations have demonstrated that solvent-driven changes in charge separation can be coupled to bond-length alternation and large variations in the NLO response [13, 15], whereas explicit, semicontinuum, and QM/MM treatments show that local solute–solvent interactions may further modulate the response beyond a purely bulk dielectric description [16, 19]. These observations motivate the present comparison of fixed-geometry and solvent-relaxed calculations for two anthracene-based chalcones and require that continuum-model predictions be interpreted without attributing them to specific first-shell interactions that are not explicitly represented.

In this context, we present a systematic theoretical investigation of two structurally related anthracene-based chalcone derivatives: ANT–IND (Structure I), bearing an indole donor, and ANT–DMNAF (Structure II), bearing a 4-(dimethylamino)naphthyl donor. The choice of these two structures allows a direct comparison between distinct donor environments within the same anthracene–enone framework. Using CAM-B3LYP/6–31 + G(d,p) with the SMD solvation model across six media (the gas phase plus five solvents), we characterize: (i) ground-state molecular geometry and structural changes upon solvation; (ii) frontier molecular orbital energies and electronic descriptors; (iii) UV–Vis absorption spectra and excited-state character; and (iv) static first hyperpolarizabilities and polarizabilities. The GasGeom/SolvGeom comparison is used to assess the sensitivity of the calculated response to solvent-field polarization and geometry relaxation without treating these computational protocols as experimentally separable contributions.

## Computational methodology

### Molecular structures and initial geometries

**Fig. 1:**
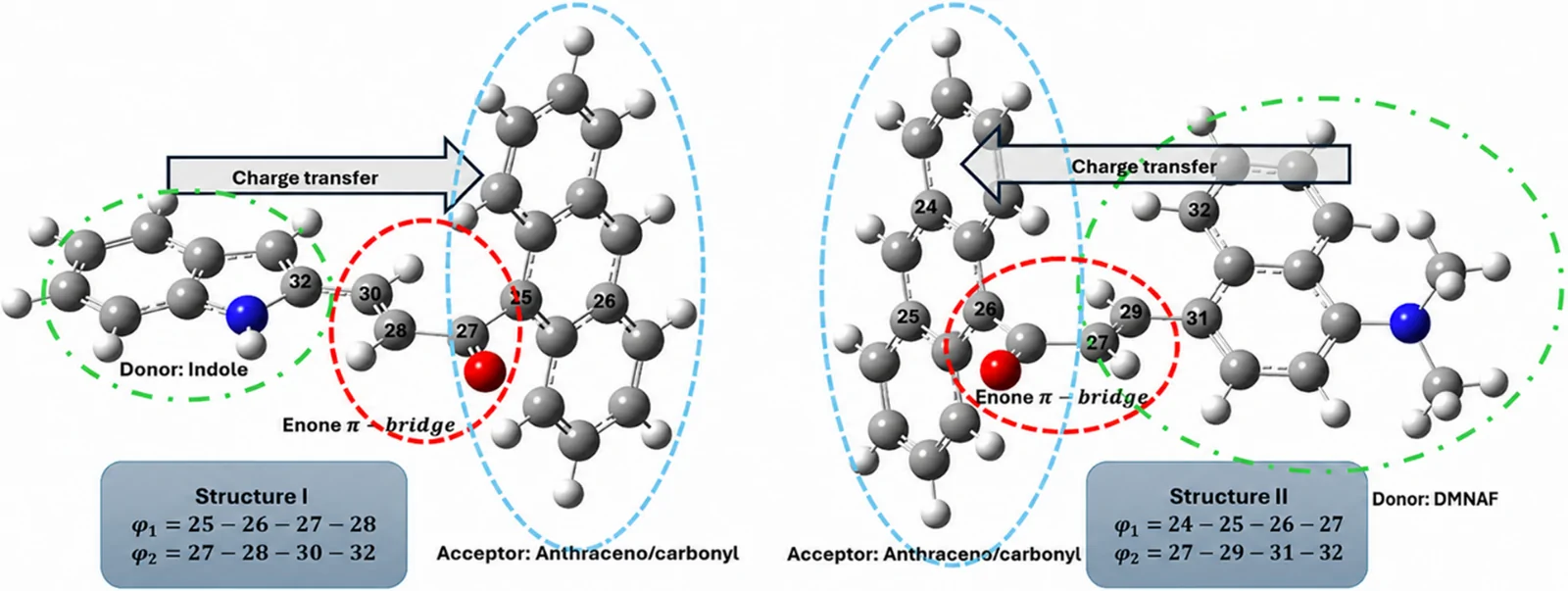
Optimized molecular structures of ANT–IND (Structure I, left) and ANT–DMNAF (Structure II, right). The annotated donor–π–acceptor framework highlights the indole and 4-(dimethylamino)naphthyl donor regions, respectively, the central α,β-unsaturated carbonyl (enone) π-bridge, and the anthracenyl acceptor fragment. Atom-number sequences used to define the principal torsional coordinates (φ₁ and φ₂) are indicated in the blue boxes. Final structures correspond to the fully relaxed CAM-B3LYP/6–31 + G(d,p) geometries

The two chalcone derivatives investigated were Structure I (ANT–IND), a 27-heavy-atom system in which the indolyl fragment acts as the electron-donor moiety, and Structure II (ANT–DMNAF), a 31-heavy-atom system containing a 4-(dimethylamino)naphthyl donor fragment [9]. In both molecules, the α,β-unsaturated carbonyl bridge provides the main π-conjugated linkage, while the anthracenyl fragment extends the conjugated framework. Initial geometries were constructed and subjected to preliminary optimization using the B3LYP exchange–correlation functional [20, 21] with the def2-SVP basis set [22] and the Grimme D3 dispersion correction with Becke–Johnson damping (D3BJ) [23]. D3BJ was used only at this preliminary stage to obtain physically reasonable starting conformations for the extended aromatic frameworks, where intramolecular dispersion can influence nonbonded distances and relative fragment orientations; the BJ damping form provides a well-behaved short-range treatment of the D3 correction. All geometries used for the reported electronic, excited-state, and NLO properties were subsequently fully and unconstrainedly reoptimized at CAM-B3LYP/6–31 + G(d,p) [24–26], in the gas phase or with SMD as appropriate. Thus, the D3BJ-corrected structures served only as starting geometries and do not directly define the final geometries or NLO values reported here. No geometric constraints were imposed in the subsequent optimizations: all atoms were fully relaxed. A systematic conformational search or torsional scan was not performed; therefore, the analysis is restricted to the selected E-configured conformer used consistently throughout the study. The optimized molecular structures of ANT–IND and ANT–DMNAF are shown in Fig. 1. All calculations were performed with Gaussian 16 [27, 28], and molecular electrostatic potential maps and frontier molecular orbital visualizations were generated using Multiwfn [29, 30].

### Geometry optimization and frequency analysis

Full geometry optimizations and harmonic frequency calculations were performed at the CAM-B3LYP/6–31 + G(d,p) level using the range-separated Coulomb-attenuating method (CAM-B3LYP) [28, 31], which is well suited to systems with significant charge-transfer character and long-range exchange effects relevant to NLO properties [31, 32]. The SCF convergence criterion was Tight, supplemented by the XQC option, and an ultrafine integration grid was used throughout. Geometry optimizations were carried out both in the gas phase and in each of the five solvents using the SMD continuum solvation model [32, 33]. Stationary-point character was confirmed by the absence of imaginary vibrational frequencies in all cases (NImag = 0; lowest real frequency > 8 cm⁻^1^).

### Solvation model

The solvent environment was modeled using the Solvation Model based on Density (SMD) of Marenich, Cramer, and Truhlar [33], which combines a self-consistent electrostatic reaction field with parametrized non-electrostatic contributions. Five solvents were selected to span a broad range of dielectric constants: n-hexane (*ε* = 1.88), toluene (*ε* = 2.37), dichloromethane (DCM, *ε* = 8.93), methanol (*ε *= 32.61), and dimethylsulfoxide (DMSO, *ε* = 46.83). Together with the gas phase, these five solvents define the six media considered in this work. SMD was applied both during solvent-phase geometry optimizations and in the subsequent electronic-response calculations, including μ, αiso, and β∥. It was selected as a consistent self-consistent continuum environment across chemically different solvents, so that solvent-induced polarization of the solute electron density and solvent-relaxed geometries could be treated within one protocol. We emphasize, however, that SMD was developed and parameterized primarily as a general solvation model, with particular emphasis on solvation thermodynamics, rather than specifically for nonlinear optical response properties [33]. Accordingly, the NLO quantities reported here should be regarded as continuum-model predictions. Specific first-shell interactions, hydrogen bonding, and configurational fluctuations are not represented explicitly; previous NLO studies using continuum, semicontinuum, explicit-solvent, and QM/MM descriptions show that such local interactions can alter the magnitude of the calculated response [12–19]. NLO calculations were evaluated under two protocols: (1) GasGeom, in which the molecular geometry is kept at the gas-phase optimum while the electronic structure is evaluated in the target solvent field; and (2) SolvGeom, in which the geometry is optimized in the target solvent before the NLO calculation. Their difference is used only as a computational measure of sensitivity to geometry relaxation within the SMD model; it is not an experimentally measurable separation of electronic and geometric contributions.

### Electronic excited-state calculations

Vertical excitation energies, oscillator strengths, and transition compositions were computed using TD-DFT [34, 35] at the CAM-B3LYP/6–31 + G(d,p) level, requesting 30 excited states. TD-DFT calculations were performed for both GasGeom and SolvGeom protocols in all six media (gas phase plus five solvents) for both structures. CAM-B3LYP was selected because range-separated hybrids provide a more appropriate description of long-range charge-transfer excitations than many global hybrids in donor − π − acceptor molecules [32]. For the most intense state reported for each medium, the dominant one-electron configurations were extracted directly from the Gaussian TD-DFT expansion coefficients. For these closed-shell singlet calculations, the percentage weight of a configuration was evaluated as $2{c}_{i}^{2} \times 100$, where ${c}_{i}$ is the printed excitation coefficient; the largest contributions are summarized in Table 5. UV–Vis absorption spectra were simulated by broadening each computed stick spectrum with a Gaussian function of HWHM = 0.20 eV.

### Nonlinear optical properties

Static first hyperpolarizabilities and isotropic polarizabilities were computed analytically using the Polar keyword in Gaussian 16. The dipole-oriented first hyperpolarizability ${\beta}_{\parallel }$ is defined as:

$${\beta}_{\parallel }=\left(\frac{3}{5}\mu \right)\sum_{i}{\mu}_{i}{\beta}_{i} ,$$

where ${\beta}_{i}={\beta}_{iii}+{\beta}_{ijj}+{\beta}_{ikk}$ (summed over all Cartesian components i,j,k). The isotropic polarizability is defined as:

$${\alpha}_{iso}=\frac{1}{3}({\alpha}_{xx}+{\alpha}_{yy}+{\alpha}_{zz}).$$

Values are reported in atomic units (a.u.) and converted to cgs units using 1 a.u. = 8.6393 × 10–33 esu. The Cartesian βᵢ component with the largest absolute magnitude identifies the dominant direction of the calculated static second-order response in the adopted molecular coordinate frame. It does not, by itself, determine the microscopic direction of intramolecular charge transfer, because β is a response tensor that contains contributions from the full electronic structure and excited-state manifold. Any relationship between a dominant β component and a donor–π–acceptor axis is therefore discussed only as an orientation of the nonlinear response and not as an independent proof of a specific charge-transfer pathway. Because first hyperpolarizabilities can be more basis-set sensitive than geometries or orbital gaps, a representative larger-basis validation was performed for Structure II in methanol. Single-point CAM-B3LYP/6–311 + + G(2d,2p)/SMD calculations [25–27, 29] were carried out at both the GasGeom and SolvGeom Cartesian geometries used in the 6–31 + G(d,p) calculations, without reoptimization, so that the basis set was the only methodological variable. The resulting deviations in μ, αiso, and ${\beta}_{\parallel }$ are reported in Sect. "Structure II (ANT–DMNAF)" and Table 8.

## Results and discussion

### Ground-state molecular geometry and structural changes upon solvation

Table 1 summarizes the key structural descriptors of Structure I (ANT–IND) in the gas phase and five solvents. The selected bond lengths, C=O (carbonyl), $C\alpha -C\beta$ (vinyl bridge), and Cα-C(ar) (conjugated single bond to the anthracenyl fragment), characterize changes in bond-length alternation (BLA) along the conjugated pathway, while the ground-state dipole moment μ provides a complementary measure of solvent-dependent polarization.

**Table 1:** Optimized structural parameters of Structure I (ANT–IND), CAM-B3LYP/6–31 + G(d,p), with SMD for solvated media. Bond lengths are in $\mathrm{\mathring{A}}$ and μ in D; the enone torsion remains < 3° in all media

**Medium (** ${\boldsymbol{\varepsilon}}$**)**	C=O $\left(\boldsymbol{\mathrm{\mathring{A}}}\right)$	${C}_{\alpha }-{C}_{\beta }$ $\left(\boldsymbol{\mathrm{\mathring{A}} }\right)$	${C}_{\alpha }-C(ar)$ $\left(\boldsymbol{\mathrm{\mathring{A}} }\right)$	${\boldsymbol{\mu}}({\boldsymbol{D}})$
Gas (1.0)	1.2224	1.3455	1.4707	3.024
n-hexane (1.88)	1.2245	1.3462	1.4682	3.315
Toluene (2.37)	1.2252	1.3465	1.4674	3.403
DCM (8.93)	1.2286	1.3476	1.4635	3.837
DMSO (46.83)	1.2288	1.3475	1.4629	3.849
Methanol (32.61)	1.2354	1.3496	1.4559	4.846

In the gas phase, the carbonyl bond length is 1.2224 $\mathrm{\mathring{A}}$. Solvation produces small but systematic, model-dependent changes rather than a monotonic dependence on the dielectric constant. The C=O bond increases to 1.2288 $\mathrm{\mathring{A}}$ in DMSO and reaches its largest value, 1.2354 $\mathrm{\mathring{A}}$, in methanol even though methanol has a lower dielectric constant than DMSO. At the same time, Cα-C(ar) shortens from 1.4707 $\mathrm{\mathring{A}}$ in the gas phase to 1.4559 $\mathrm{\mathring{A}}$ in methanol, while $C\alpha -C\beta$ increases from 1.3455 to 1.3496 $\mathrm{\mathring{A}}$. These changes are consistent with a reduction of BLA and enhanced delocalization along the conjugated pathway [35, 36], but the methanol/DMSO reversal shows that the structural response is not governed by ε alone. Within SMD, non-electrostatic solvent parameters and the resulting optimized geometry also contribute to the observed trend.

Structure II (ANT–DMNAF) is more polar than Structure I in the gas phase (μ= 4.944 D versus 3.024 D). Using a consistent CAM-B3LYP/6–31 + G(d,p) ground-state orbital analysis associated with the TD-DFT calculations, the gas-phase HOMO–LUMO gap is 5.737 eV for Structure II and 5.638 eV for Structure I. Table 2 extends the comparison for Structure II across all media and shows that the dipole moment increases substantially upon solvation, while the orbital gap changes more moderately and non-monotonically.

**Table 2:** Solvent-dependent ground-state electronic descriptors for Structure II (ANT–DMNAF). Solvated rows use solvent-optimized (SolvGeom) geometries at CAM-B3LYP/6–31 + G(d,p)/SMD; the gas row uses the gas-phase optimized geometry. HOMO and LUMO energies are the ground-state Kohn–Sham orbital energies from the same calculations used as the TD-DFT reference

Medium (ε)	μ (D)	HOMO (eV)	LUMO (eV)	Gap (eV)
Gas (1.0)	4.944	− 6.790	− 1.053	5.737
n-hexane (1.88)	5.625	− 6.722	− 0.989	5.733
Toluene (2.37)	5.837	− 6.717	− 0.987	5.730
DCM (8.93)	6.931	− 6.730	− 1.064	5.666
DMSO (46.83)	7.127	− 6.732	− 1.062	5.670
Methanol (32.61)	8.182	− 6.757	− 1.206	5.551

**Fig. 2:**
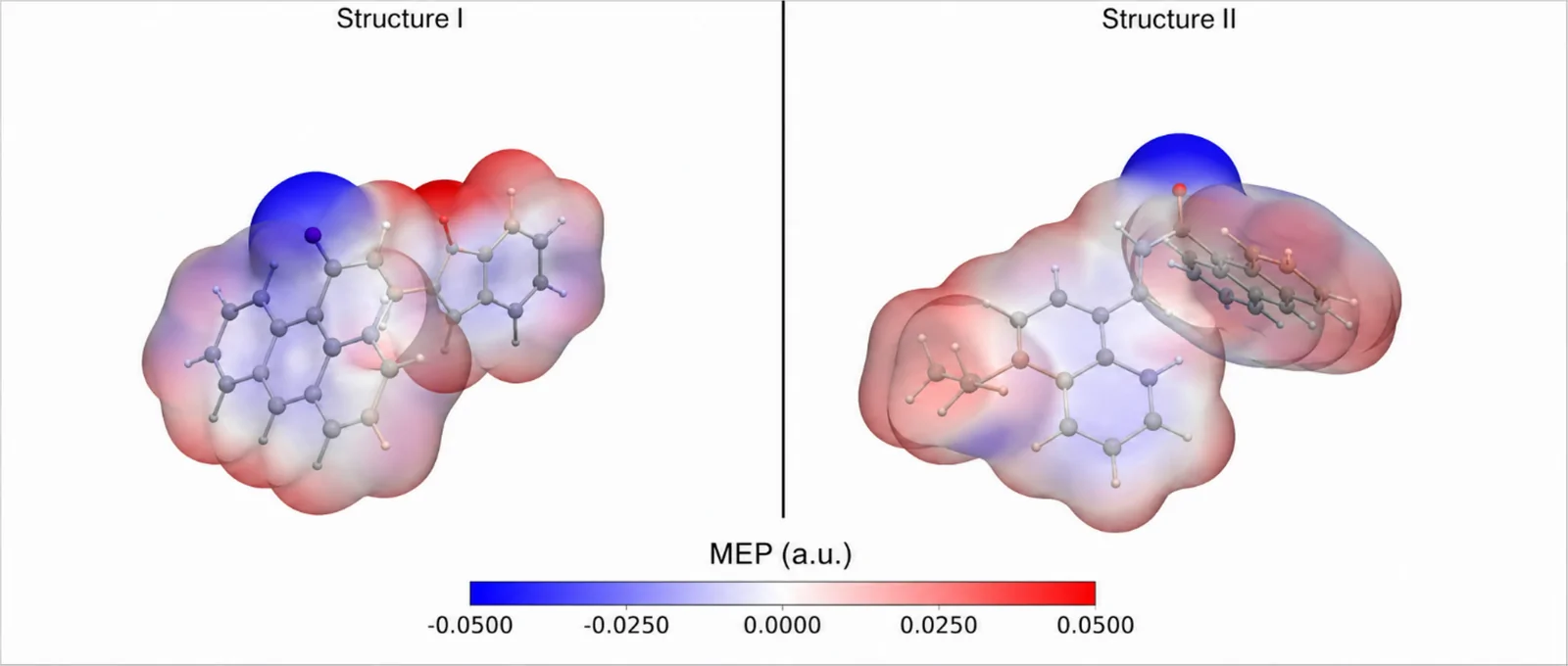
Molecular electrostatic potential (MEP) surfaces of ANT–IND (Structure I, left) and ANT–DMNAF (Structure II, right) in the gas phase. The electrostatic potential is mapped onto the molecular electron-density surface using the common scale shown below the structures (− 0.0500 to + 0.0500 a.u.); blue and red regions correspond to the negative and positive ends of the displayed potential scale, respectively. The surfaces were generated from the CAM-B3LYP/6–31 + G(d,p) electron densities using Multiwfn

The molecular electrostatic potential surfaces of both chalcone derivatives in the gas phase are presented in Fig. 2, illustrating the spatial distribution of electron-rich and electron-poor regions.

Tables 3 and 4 summarize the most intense TD-DFT transitions for Structures I and II at the solvent-optimized (SolvGeom) geometries in the five solvents, together with the corresponding gas-phase results. The most intense transitions fall in the UV region (230–250 nm), characteristic of extended aromatic systems containing anthracene [9]. The HOMO and LUMO entries report the ground-state Kohn–Sham orbital energies from these same CAM-B3LYP/6–31 + G(d,p) calculations, so a single consistent protocol is used throughout Tables 3 and 4. Importantly, the configuration analysis in Table 5 shows that these intense states are not dominated by a simple HOMO → LUMO excitation; rather, they are multi-orbital states involving several frontier and near-frontier orbitals.

**Table 3:** TD-DFT absorption data for Structure I (ANT–IND), CAM-B3LYP/6–31 + G(d,p), with SMD for solvated media (SolvGeom). Configuration weights are given in Table 5

**Medium (** ${\boldsymbol{\varepsilon}}$**)**	**State**	${{\boldsymbol{\lambda}}}_{{\boldsymbol{m}}{\boldsymbol{a}}{\boldsymbol{x}}}$**(nm)**	**E (eV)**	${\boldsymbol{f}}$	**HOMO (eV)**	**LUMO (eV)**	$\boldsymbol{\Delta }{\boldsymbol{E}}$**(eV)**
Gas (1.0)	S11	231.2	5.363	1.189	− 6.862	− 1.223	5.638
n-hexane (1.88)	S9	243.2	5.099	1.987	− 6.778	− 1.136	5.642
Toluene (2.37)	S9	245.9	5.043	2.154	− 6.767	− 1.123	5.644
DCM (8.93)	S9	244.3	5.076	2.155	− 6.772	− 1.127	5.646
DMSO (46.83)	S9	244.0	5.082	2.162	− 6.782	− 1.106	5.676
Methanol (32.61)	S10	241.5	5.134	1.300	− 6.804	− 1.257	5.547

**Table 4:** TD-DFT absorption data for Structure II (ANT–DMNAF), CAM-B3LYP/6–31 + G(d,p), with SMD for solvated media (SolvGeom). Configuration weights are given in Table 5

**Medium (** ${\boldsymbol{\varepsilon}}$**)**	**State**	${{\boldsymbol{\lambda}}}_{{\boldsymbol{m}}{\boldsymbol{a}}{\boldsymbol{x}}}$**(nm)**	**E (eV)**	${\boldsymbol{f}}$	**HOMO (eV)**	**LUMO (eV)**	$\boldsymbol{\Delta }{\boldsymbol{E}}$**(eV)**
Gas (1.0)	S16	230.5	5.378	1.559	− 6.790	− 1.053	5.737
n-hexane (1.88)	S10	243.8	5.085	1.730	− 6.722	− 0.989	5.733
Toluene (2.37)	S10	246.5	5.030	1.930	− 6.717	− 0.987	5.730
DCM (8.93)	S11	244.9	5.063	1.583	− 6.730	− 1.064	5.666
DMSO (46.83)	S10	244.7	5.067	1.355	− 6.732	− 1.062	5.670
Methanol (32.61)	S13	241.8	5.127	2.139	− 6.757	− 1.206	5.551

**Table 5:** Dominant TD-DFT configurations for the most intense states reported in Tables 3 and 4. H and L denote HOMO and LUMO, respectively. Percentages were calculated as $2{c}_{i}^{2}\times 100$ from the Gaussian closed-shell singlet excitation coefficients. The largest contributions are listed; smaller terms are omitted for clarity

Structure	Medium/state	Dominant configurations (weight)
I	Gas/S11	H → L + 2 (33.6%); H − 3 → L + 1 (30.3%); H → L + 6 (13.7%)
I	n-hexane/S9	H → L + 2 (44.5%); H − 3 → L + 1 (42.6%); H − 4 → L + 1 (6.4%)
I	Toluene/S9	H → L + 2 (47.0%); H − 3 → L + 1 (45.8%)
I	DCM/S9	H → L + 2 (47.3%); H − 3 → L + 1 (46.2%)
I	DMSO/S9	H → L + 2 (47.2%); H − 3 → L + 1 (46.8%)
I	Methanol/S10	H → L + 2 (31.8%); H − 3 → L + 1 (28.5%); H − 4 → L + 1 (21.2%); H → L + 5 (11.8%)
II	Gas/S16	H − 2 → L + 1 (30.6%); H → L + 3 (29.9%); H − 4 → L (9.3%); H − 1 → L + 4 (7.3%)
II	n-hexane/S10	H → L + 3 (44.3%); H − 2 → L + 1 (41.9%)
II	Toluene/S10	H → L + 3 (44.8%); H − 2 → L + 1 (44.0%)
II	DCM/S11	H − 2 → L + 1 (31.6%); H → L + 3 (23.4%); H − 1 → L + 3 (16.8%); H − 4 → L (8.6%)
II	DMSO/S10	H − 1 → L + 3 (24.6%); H − 3 → L + 1 (19.0%); H − 2 → L + 1 (14.6%); H − 4 → L (13.0%)
II	Methanol/S13	H − 2 → L + 1 (37.4%); H − 1 → L + 3 (36.5%); H → L + 3 (11.0%); H − 3 → L + 1 (7.9%)

The configuration weights answer directly whether the intense states in Tables 3 and 4 are HOMO → LUMO transitions: they are not. For Structure I, the solvent-phase S9 band is dominated by H → L + 2 and H − 3 → L + 1 contributions, each contributing roughly 43–47% in n-hexane, toluene, DCM, and DMSO. In methanol, S10 becomes more strongly mixed, with H → L + 2 (31.8%), H − 3 → L + 1 (28.5%), H − 4 → L + 1 (21.2%), and H → L + 5 (11.8%). Structure II is similarly multi-orbital: the principal solvent-phase bands are dominated mainly by H → L + 3 and H − 2 → L + 1, while DCM and DMSO show stronger mixing with additional H − 1 and H − 3 contributions. Methanol S13 contains H − 2 → L + 1 (37.4%) and H − 1 → L + 3 (36.5%) as its two largest terms. Figure 4 is therefore retained only to illustrate the frontier-orbital redistribution associated with the donor -π- acceptor framework; it should not be read as a direct orbital assignment of the intense 230–250 nm bands. The configuration analysis establishes the multi-orbital nature of these states, but the Gaussian expansion coefficients alone do not provide a quantitative spatial measure of locally excited versus charge-transfer character. A rigorous spatial decomposition would require natural-transition-orbital, hole–electron, or transition-density analysis. We consequently avoid assigning a unique microscopic charge-transfer direction to these states from HOMO/LUMO plots alone.

Both structures display a bathochromic shift upon solvation: the principal absorption band moves from approximately 231 nm in the gas phase to roughly 243–246 nm in most solvents. From the tabulated excitation energies, the decrease relative to the gas phase spans approximately 0.23–0.32 eV for Structure I and 0.25–0.35 eV for Structure II (about 0.23–0.35 eV over the complete dataset). Thus, the previously quoted 0.28–0.33 eV interval was too narrow. Within the solvent set, the wavelength variation is modest, with toluene giving the longest-wavelength maximum and methanol a shorter-wavelength maximum than several of the other solvents.

**Fig. 3:**
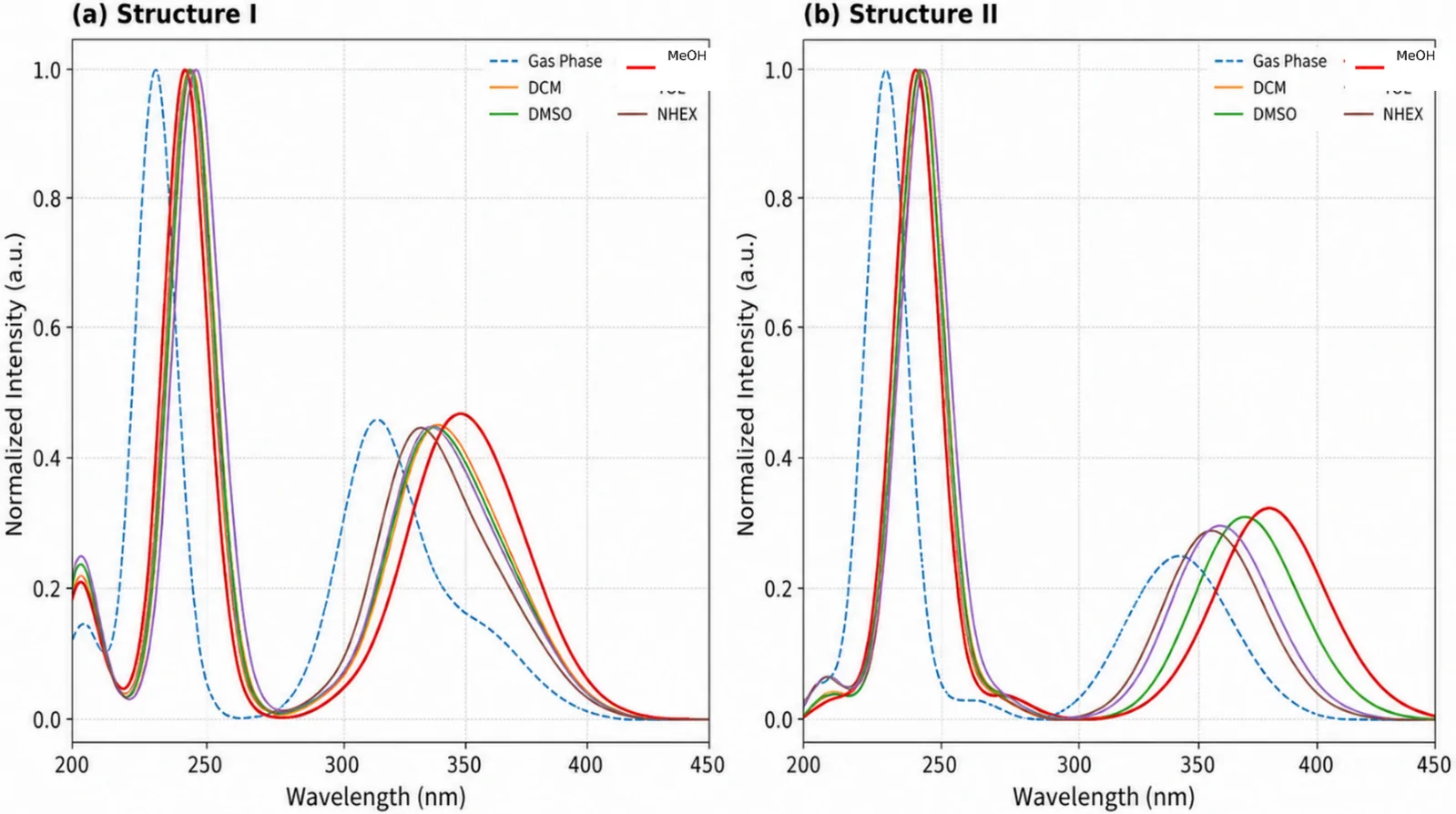
TD-DFT simulated UV–Vis absorption spectra of **a** ANT–IND (Structure I) and **b** ANT–DMNAF (Structure II) in the gas phase, n-hexane, toluene, DCM, DMSO, and methanol. Solvent spectra were calculated at the corresponding solvent-relaxed geometries (SolvGeom), whereas the gas-phase curve uses the gas-phase optimized geometry. CAM-B3LYP/6–31 + G(d,p) was employed, with SMD for solvated media and 30 singlet excited states. Each spectrum was independently normalized to its maximum intensity, and Gaussian broadening with HWHM = 0.20 eV was applied to the calculated transitions

A notable feature is the behavior in methanol. For Structure I, the most intense transition in methanol is S10 at 241.5 nm with f= 1.300, lower than in DCM or DMSO (f≈ 2.15–2.16). For Structure II, methanol yields the largest oscillator strength in the dataset (f= 2.139, S13), whereas DMSO gives f= 1.355. Because Table 1 shows that the enone core remains essentially planar (torsion < 3°), the opposite oscillator-strength behavior is not attributed to a loss of enone planarity. A more conservative interpretation is that solvent-dependent electronic polarization together with subtle geometry changes outside the enone core modifies the orbital mixing differently in the two donor environments. No dedicated conformational scan of the donor torsions was performed, so a specific torsional mechanism is not assigned here. The simulated TD-DFT UV–Vis absorption spectra are shown in Fig. 3.

**Fig. 4:**
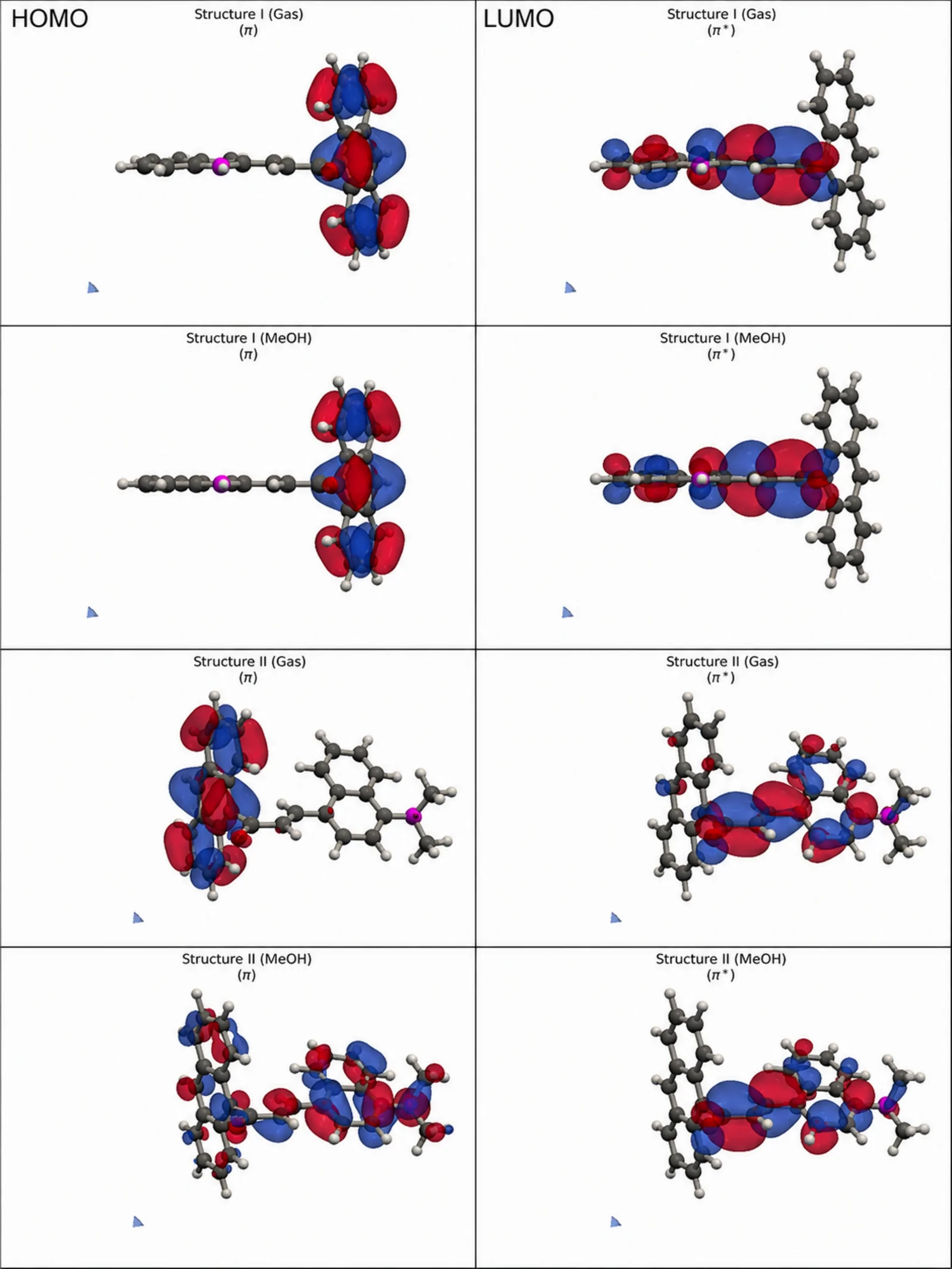
Frontier molecular orbitals (HOMO and LUMO) of ANT–IND (Structure I) and ANT–DMNAF (Structure II) in the gas phase and methanol. The panels compare the orbital distributions obtained from CAM-B3LYP/6–31 + G(d,p) calculations at the corresponding optimized geometries, with SMD used for methanol. Red and blue isosurfaces denote opposite orbital phases. These plots are provided as qualitative frontier-orbital context and are not used to assign the intense 230–250 nm absorption bands to a single HOMO → LUMO transition

The HOMO–LUMO gap shows a mild, non-monotonic solvent dependence. Using the consistent orbital energies reported in Tables 3 and 4, ΔE spans approximately 5.547–5.737 eV. The smallest values occur in methanol: 5.547 eV for Structure I and 5.551 eV for Structure II. These values are smaller than those in DMSO despite the larger dielectric constant of DMSO, again indicating that the SMD response is not governed by ε alone. The frontier molecular orbitals of both structures in the gas phase and methanol are displayed in Fig. 4 as qualitative context for the donor–π–acceptor electronic distribution. Because the intense states summarized in Tables 3, 4, and 5 involve higher occupied and virtual orbitals, Fig. 4 is not used to claim that those absorption maxima correspond to HOMO → LUMO charge transfer.

### Nonlinear optical properties

Tables 6 and 7 present the static NLO properties, dipole moment μ, isotropic polarizability αiso, and dipole-oriented first hyperpolarizability ${\beta}_{\parallel }$, for Structures I and II across the gas phase and five solvents. GasGeom and SolvGeom are retained as two computational protocols for evaluating sensitivity to geometry relaxation. The SolvGeom results are used for the solvent-relaxed solution-phase comparisons, whereas GasGeom values are treated as fixed-geometry diagnostic calculations rather than experimentally measurable solvent responses.

**Table 6:** Static NLO properties of Structure I (ANT–IND), CAM-B3LYP/6–31 + G(d,p), with SMD for solvated media. $\Delta {\beta}_{\parallel }$ is relative to the gas reference (2645 a.u.)

**Solvent**	**Geom**	${\boldsymbol{\mu}}({\boldsymbol{D}})$	${{\boldsymbol{\alpha}}}_{{\boldsymbol{i}}{\boldsymbol{s}}{\boldsymbol{o}}}$**(a.u.)**	${\beta}_{\parallel }$**(a.u.)**	${\beta}_{\parallel }$**(** ${\times 10}^{-30}$** esu)**	**Δ** ${\beta}_{\parallel }$	**Dom. comp**
Gas	Gas	3.024	337.24	2645	22.85	—	βz
n-hexane	Gas	3.274	389.52	3882	33.53	+ 47%	βz
n-hexane	Solv	3.315	390.22	3947	34.10	+ 49%	βz
Toluene	Gas	3.343	406.80	4298	37.13	+ 63%	βz
Toluene	Solv	3.403	407.76	4389	37.92	+ 66%	βz
DCM	Gas	3.684	478.72	6155	53.18	+ 133%	βz
DCM	Solv	3.837	481.31	6441	55.64	+ 143%	βz
DMSO	Gas	3.663	509.33	6651	57.46	+ 151%	βz
DMSO	Solv	3.849	512.17	6995	60.43	+ 165%	βz
Methanol	Gas	4.413	514.46	8100	69.98	+ 206%	βz
Methanol	Solv	4.846	519.99	8886	76.77	+ 236%	βz

**Table 7:** Static NLO properties of Structure II (ANT–DMNAF), CAM-B3LYP/6–31 + G(d,p), with SMD for solvated media. $\Delta {\beta}_{\parallel }$ is relative to the gas reference (2393 a.u.)

**Solvent**	**Geom**	${\boldsymbol{\mu}}({\boldsymbol{D}})$	${{\boldsymbol{\alpha}}}_{{\boldsymbol{i}}{\boldsymbol{s}}{\boldsymbol{o}}}$**(a.u.)**	${\beta}_{\parallel }$**(a.u.)**	${\beta}_{\parallel }$**(** ${\times 10}^{-30}$** esu)**	**Δ** ${\beta}_{\parallel }$	**Dom. comp**
Gas	Gas	4.944	376.47	2393	20.68	—	βz
n-hexane	Gas	5.456	435.21	3566	30.81	+ 49%	βz
n-hexane	Solv	5.625	436.96	3782	32.67	+ 58%	βz
Toluene	Gas	5.606	454.84	3969	34.29	+ 66%	βz
Toluene	Solv	5.837	457.25	4282	36.99	+ 79%	βz
DCM	Gas	6.249	537.39	5090	43.97	+ 113%	βz
DCM	Solv	6.931	545.80	6422	55.48	+ 168%	βz
DMSO	Gas	6.376	573.93	6338	54.75	+ 165%	βz
DMSO	Solv	7.127	583.13	7936	68.56	+ 232%	βz
Methanol	Gas	7.037	576.97	7553	65.26	+ 216%	βz
Methanol	Solv	8.182	590.97	10,269	88.71	+ 329%	βz

#### Structure I (ANT–IND)

In the gas phase, Structure I gives ${\beta}_{\parallel }$ = 2645 a.u. (22.85 $\times {10}^{-30}$ esu), comparable in magnitude to anthracene-containing donor -π- acceptor chalcones reported in the literature [30, 37]. For the solvent-relaxed geometries (SolvGeom), the total increase relative to the gas-phase reference is + 49% in n-hexane, + 66% in toluene, + 143% in DCM, + 165% in DMSO, and + 236% in methanol. These SolvGeom percentages are the values used to discuss the overall solvent-dependent response. The corresponding GasGeom calculations are retained only as fixed-geometry diagnostics of the effect of placing the gas-phase structure in the solvent field.

The GasGeom-to-SolvGeom difference quantifies how sensitive the computed ${\beta}_{\parallel }$ is to geometry relaxation within the same solvent model. For Structure I, this change is about + 1.7% in n-hexane, + 2.1% in toluene, + 4.6% in DCM, + 5.2% in DMSO, and + 9.7% in methanol, each relative to the corresponding GasGeom value. These increments are positive in the present calculations, but they are not classified as a secondary physical contribution because GasGeom and SolvGeom are alternative computational protocols rather than experimentally separable response components. The accompanying BLA changes are consistent with the trend but do not by themselves establish a unique causal partition.

In all media, the z-directed contribution remains the largest Cartesian contribution to the static hyperpolarizability in the adopted coordinate system. Because the z axis is approximately aligned with the molecular donor -π- acceptor framework, the strongest calculated second-order response is therefore oriented predominantly along this molecular direction. However, the dominant tensor component alone does not establish the microscopic charge-transfer pathway, and no one-to-one identification between β and the direction of intramolecular charge transfer is made here.

#### Structure II (ANT–DMNAF)

Structure II has a gas-phase ${\beta}_{\parallel }$ of 2393 a.u. (20.68 $\times {10}^{-30}$ esu), slightly lower than the 2645 a.u. value of Structure I despite its stronger dimethylamino donor environment. A simple donor-strength argument is therefore insufficient for the gas-phase ordering. The consistent CAM-B3LYP/6–31 + G(d,p) orbital data also show a somewhat larger gas-phase HOMO–LUMO gap for Structure II (5.737 eV) than for Structure I (5.638 eV), which is compatible with a smaller ${\beta}_{\parallel }$ within the qualitative two-state picture [36, 38]. Because no exhaustive conformational scan was performed, the gas-phase difference is not assigned to a specific donor torsion.

Structure II shows a stronger solvent response after full geometry relaxation. The SolvGeom values are 3782 a.u. in n-hexane (+ 58%), 4282 a.u. in toluene (+ 79%), 6422 a.u. in DCM (+ 168%), 7936 a.u. in DMSO (+ 232%), and 10269 a.u. in methanol (+ 329%) relative to the 2393 a.u. gas-phase reference. The methanol value is the largest in the dataset. The dipole moment also increases from 4.944 D in the gas phase to 8.182 D at the methanol-optimized geometry, consistent with stronger stabilization of a polarized electronic distribution in the continuum model. GasGeom values are retained as fixed-geometry diagnostic results and are not used as the total solution-phase enhancement.

**Fig. 5:**
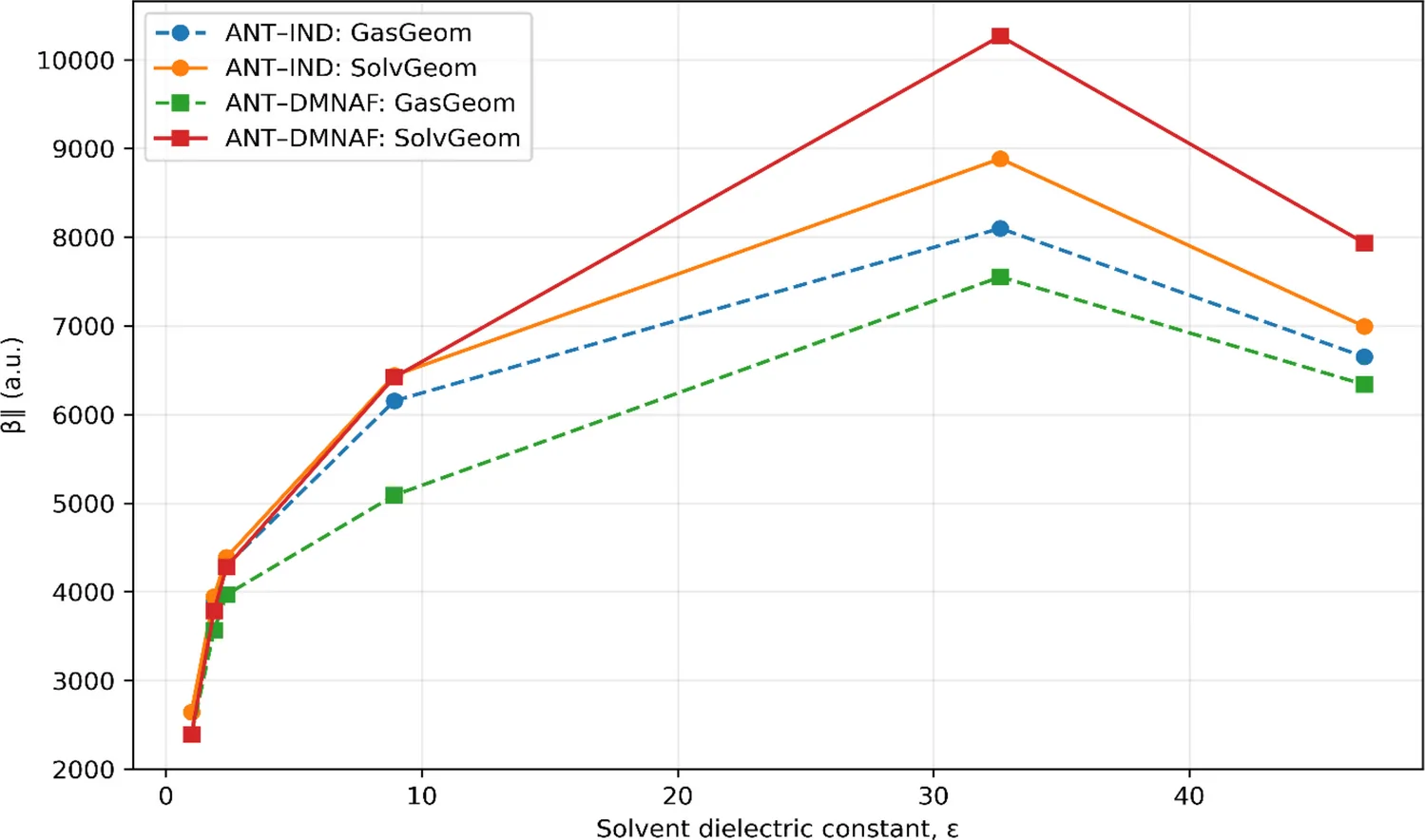
Static dipole-oriented first hyperpolarizability, β∥ (a.u.), as a function of solvent dielectric constant for ANT–IND and ANT–DMNAF. Dashed GasGeom curves correspond to fixed gas-phase geometries evaluated in each solvent field, whereas solid SolvGeom curves correspond to geometries fully relaxed in the respective solvent and are used for the equilibrium-in-continuum comparison. Values were obtained at CAM-B3LYP/6–31 + G(d,p), using SMD for the solvated calculations; the gas-phase reference is shown at *ε* = 1

The comparison between Structures I and II reveals a crossover in NLO performance as the medium changes. Structure I is slightly higher in the gas phase and low-polarity media, whereas Structure II becomes larger in DCM, DMSO, and methanol after solvent relaxation. The GasGeom-to-SolvGeom change is also more pronounced for Structure II: in methanol it is 2716 a.u. (about + 36% relative to GasGeom), compared with 786 a.u. (about + 9.7%) for Structure I. This difference demonstrates greater geometry sensitivity of the computed response for Structure II within the present protocol, but it is not interpreted as an experimentally isolated geometric contribution. The calculated ${\beta}_{\parallel }$ values plotted against dielectric constant are summarized in Fig. 5.

A representative basis-set sensitivity test was carried out for Structure II in methanol by repeating the GasGeom and SolvGeom single-point NLO calculations with 6–311 + + G(2d,2p), while keeping the Cartesian geometries and all other computational settings unchanged. The larger basis changes the GasGeom values by + 0.15% for μ, + 3.58% for ${\alpha}_{iso}$, and − 1.99% for ${\beta}_{\parallel }$, and the SolvGeom values by + 0.38%, + 3.53%, and − 1.93%, respectively (Table 8).

**Table 8:** Basis-set sensitivity test for Structure II (ANT–DMNAF) in methanol. The 6–311 + + G(2d,2p) calculations use exactly the same GasGeom and SolvGeom Cartesian geometries as the 6–31 + G(d,p) calculations. Deviation (%) = 100[Xlarge − Xsmall]/Xsmall

**Property**	**Geometry**	**6–31 + G(d,p)**	**6–311 + + G(2d,2p)**	**Deviation (%)**
${\boldsymbol{\mu}}({\boldsymbol{D}})$	GasGeom	7.0365	7.0471	+ 0.15
${\boldsymbol{\mu}}({\boldsymbol{D}})$	SolvGeom	8.1817	8.2131	+ 0.38
${{\boldsymbol{\alpha}}}_{{\boldsymbol{i}}{\boldsymbol{s}}{\boldsymbol{o}}}({\boldsymbol{a}}.{\boldsymbol{u}}.)$	GasGeom	576.969	597.650	+ 3.58
${{\boldsymbol{\alpha}}}_{{\boldsymbol{i}}{\boldsymbol{s}}{\boldsymbol{o}}}({\boldsymbol{a}}.{\boldsymbol{u}}.)$	SolvGeom	590.967	611.812	+ 3.53
${{\boldsymbol{\beta}}}_{\parallel }({\boldsymbol{a}}.{\boldsymbol{u}}.)$	GasGeom	7553.38	7403.32	− 1.99
${{\boldsymbol{\beta}}}_{\parallel }({\boldsymbol{a}}.{\boldsymbol{u}}.)$	SolvGeom	10,268.8	10,070.7	− 1.93

The GasGeom-to-SolvGeom increase in ${\beta}_{\parallel }$ is essentially unchanged by the basis enlargement: 35.95% with 6–31 + G(d,p) and 36.03% with 6–311 + + G(2d,2p). Thus, for this representative high-response case, the absolute ${\beta}_{\parallel }$ values differ by only about 2% and the geometry-relaxation trend is preserved to within 0.08 percentage points. This test supports the robustness of the principal trend discussed here, while it should not be interpreted as a complete basis-set convergence study for every solvent and both structures.

### Structure–property relationships and design implications

The combined analysis of structural, spectroscopic, and NLO data allows three key structure–property relationships to be identified for the design of improved anthracene-based chalcone chromophores.

First, donor environment and solvation response. Structure II does not have the larger gas-phase β∥, yet it develops the larger solvent-relaxed ${\beta}_{\parallel }$ in DCM, DMSO, and methanol. Thus, gas-phase donor strength alone does not determine the solution-phase ordering. Because no exhaustive conformational scan was performed, the difference is not assigned uniquely to donor flexibility or to a specific torsional coordinate; it is reported as the combined electronic and geometry response of the two molecular frameworks within the SMD protocol.

Second, interpretation of the two computational protocols. The SolvGeom values are the physically relevant equilibrium-in-continuum predictions for molecules allowed to relax in the target solvent and are therefore used for the reported solution-phase percentage enhancements. GasGeom is retained only as a fixed-geometry sensitivity test. The difference between GasGeom and SolvGeom quantifies how much the computed ${\beta}_{\parallel }$ changes when the molecular geometry is relaxed under the same solvent model, but it should not be interpreted as an experimentally measurable electronic/geometric partition or as first- and second-order optical contributions. To avoid ambiguity, we therefore refer to the protocols as fixed-geometry and relaxed-geometry calculations rather than as first- and second-order approximations.

Third, solvent selection: methanol vs. DMSO. Methanol gives the largest NLO response for both structures, despite its lower dielectric constant than DMSO (*ε* = 32.61 vs. 46.83). Because the present calculations use an implicit SMD description, this result should be interpreted as a solvent-model effect combining dielectric and non-electrostatic SMD parameters rather than as direct evidence of explicit hydrogen bonding. It nevertheless motivates future explicit-solvation calculations with methanol clusters to test whether C = O···H–O interactions further amplify the response.

## Conclusions

We have presented a theoretical characterization of ANT–IND (Structure I) and ANT–DMNAF (Structure II) in six media, the gas phase and five solvents, using DFT and TD-DFT at the CAM-B3LYP/6–31 + G(d,p)/SMD level for the solvated calculations. The principal findings are summarized below.

Solvation produces computed changes in the optimized geometry of Structure I, including C=O elongation and Cα-C(ar) shortening, but these variations are not monotonic with the dielectric constant. Methanol produces the largest changes among the media examined despite having a lower ε than DMSO, emphasizing that the SMD response reflects more than dielectric polarization alone. All optimized structures are true minima with no imaginary frequencies.

The UV–Vis spectra show a bathochromic shift of about 10–16 nm for the most intense transition relative to the gas phase, with excitation-energy decreases spanning approximately 0.23–0.35 eV across the complete dataset. The opposite oscillator-strength behavior of the two structures in methanol is discussed conservatively in terms of solvent-dependent polarization and subtle geometry changes; it is not attributed to enone-planarity loss, and no specific donor-torsion mechanism is claimed without a conformational scan.

Both structures show substantial increases in solvent-relaxed ${\beta}_{\parallel }$ relative to the gas phase. For Structure I, the SolvGeom enhancement reaches + 143% in DCM, + 165% in DMSO, and + 236% in methanol; for Structure II, the corresponding values are + 168%, + 232%, and + 329%. The GasGeom/SolvGeom comparison shows that geometry relaxation changes the calculated response only modestly for Structure I but more strongly for Structure II, especially in polar media. This protocol difference is treated as a computational sensitivity analysis rather than an experimentally separable mechanistic partition.

Structure II (ANT–DMNAF) gives the highest absolute $\beta_\parallel$ in the dataset: 10,269 a.u. (88.7 × 10^-30^ esu) at the methanoloptimized geometry, a + 329% increase relative to its gas-phase value. Despite its lower gas-phase $\beta_\parallel$, ANT–DMNAF therefore shows the larger solvent-relaxed response in polar media within the present SMD model. This solvent-dependent crossover may be relevant to the design and screening of solution-processable NLO chromophores. A representative 6–311 + + G(2d,2p) validation in methanol gives $\beta_\parallel$ = 10,070.7 a.u. for SolvGeom (− 1.93% relative to 6–31 + G(d,p)) and preserves the GasGeom-to-SolvGeom increase at approximately 36%, supporting the robustness of this high-response trend within the tested basis-set enlargement.

Future work will extend this analysis to explicit methanol-cluster models to test possible hydrogen-bonding effects, frequency-dependent hyperpolarizabilities, and natural-transition-orbital or hole–electron analysis to quantify the spatial charge-transfer character of the excited states. Such extensions are particularly relevant because SMD provides a continuum description and was not specifically parameterized for NLO response observables.

## Data Availability

The computational datasets generated and analyzed during the current study, including optimized geometries, electronic descriptors, TD-DFT transition data, UV–Vis spectra, dipole moments, polarizabilities, and first hyperpolarizabilities, are available from the corresponding author upon reasonable request.
